# Exploration of oxidized phosphocholine profile in non-small-cell lung cancer

**DOI:** 10.3389/fmolb.2023.1279645

**Published:** 2024-01-15

**Authors:** Joanna Godzien, Angeles Lopez-Lopez, Julia Sieminska, Kacper Jablonowski, Karolina Pietrowska, Joanna Kisluk, Malgorzata Mojsak, Zofia Dzieciol-Anikiej, Coral Barbas, Joanna Reszec, Miroslaw Kozlowski, Marcin Moniuszko, Adam Kretowski, Jacek Niklinski, Michal Ciborowski

**Affiliations:** ^1^ Metabolomics Laboratory, Clinical Research Centre, Medical University of Bialystok, Bialystok, Poland; ^2^ Centro de Metabolómica y Bioanálisis (CEMBIO), Facultad de Farmacia, Universidad San Pablo-CEU, CEU Universities, Boadilla del Monte, Spain; ^3^ Department of Clinical Molecular Biology, Medical University of Bialystok, Bialystok, Poland; ^4^ Independent Laboratory of Molecular Imaging, Medical University of Bialystok, Bialystok, Poland; ^5^ Department of Rehabilitation, Medical University of Bialystok, Bialystok, Poland; ^6^ Department of Medical Pathomorphology, Medical University of Bialystok, Bialystok, Poland; ^7^ Department of Thoracic Surgery, Medical University of Bialystok, Bialystok, Poland; ^8^ Department of Regenerative Medicine and Immune Regulation, Medical University of Bialystok, Bialystok, Poland

**Keywords:** NSCLC, lung cancer, oxPC, oxidized phospholipids, epilipidomics

## Abstract

**Introduction:** Lung cancer is one of the most frequently studied types of cancer and represents the most common and lethal neoplasm. Our previous research on non-small cell lung cancer (NSCLC) has revealed deep lipid profile reprogramming and redox status disruption in cancer patients. Lung cell membranes are rich in phospholipids that are susceptible to oxidation, leading to the formation of bioactive oxidized phosphatidylcholines (oxPCs). Persistent and elevated levels of oxPCs have been shown to induce chronic inflammation, leading to detrimental effects. However, recent reports suggest that certain oxPCs possess anti-inflammatory, pro-survival, and endothelial barrier-protective properties. Thus, we aimed to measure the levels of oxPCs in NSCLC patients and investigate their potential role in lung cancer.

**Methods:** To explore the oxPCs profiles in lung cancer, we performed in-depth, multi-level metabolomic analyses of nearly 350 plasma and lung tissue samples from 200 patients with NSCLC, including adenocarcinoma (ADC) and squamous cell carcinoma (SCC), the two most prevalent NSCLC subtypes and COPD patients as a control group. First, we performed oxPC profiling of plasma samples. Second, we analyzed tumor and non-cancerous lung tissues collected during the surgical removal of NSCLC tumors. Because of tumor tissue heterogeneity, subsequent analyses covered the surrounding healthy tissue and peripheral and central tumors. To assess whether the observed phenotypic changes in the patients were associated with measured oxPC levels, metabolomics data were augmented with data from medical records.

**Results:** We observed a predominance of long-chain oxPCs in plasma samples and of short-chain oxPCs in tissue samples from patients with NSCLC. The highest concentration of oxPCs was observed in the central tumor region. ADC patients showed higher levels of oxPCs compared to the control group, than patients with SCC.

**Conclusion:** The detrimental effects associated with the accumulation of short-chain oxPCs suggest that these molecules may have greater therapeutic utility than diagnostic value, especially given that elevated oxPC levels are a hallmark of multiple types of cancer.

## 1 Introduction

Redox balance is one of the most important factors influencing the health status of an organism. It is crucial for proper cellular signaling and communication and is thus essential for the healthy functioning of living organisms. Disruptions in this balance result in an excess of reactive oxygen species (ROS), reducing the antioxidant potential of the cell and causing oxidative stress.

The idea of evaluating the health of an organism based on oxidative stress levels is not new. Among different oxidation products, lipids play an important role, especially since lipid peroxidation is a damaging process that can contribute to the development and progression of various diseases. Lipid oxidation has been deeply investigated not only through stable end-products, such as 4-hydroxynonenal (4-HNE) and malondialdehyde (MDA) (Zabłocka-Słowińska et al., 2019) but currently also through early oxidation products, including oxidized phospholipids (Solati et al., 2021). Oxidized phosphatidylcholines (oxPCs) are among the most thoroughly researched oxidized phospholipids and comprise a broad group of bioactive molecules that differ in structure and function. OxPCs may exist as long-chain solely (LCh-oxPCs) or as short-chain forms (SCh-oxPCs) produced by the oxidative fragmentation of esterified polyunsaturated fatty acids (PUFAs); and cyclized oxPCs (Cyc-oxPCs) form via cyclization of peroxyl radicals (Davies and Guo, 2014). LCh-oxPCs have oxidized fatty acid, usually in the *sn*-2 position of glycerol-phospholipid, containing oxidation-derived functional groups such as hydroxy, hydroperoxy, epoxy or keto. SCh-oxPCs have truncated fatty acid, usually in the *sn*-2 position of glycerol-phospholipid, containing a terminal aldehyde or carboxylic acid (Gil de la Fuente et al., 2018; Villaseñor et al., 2021).

Historically, oxPCs have been considered toxic oxidation by-products; however, current evidence acknowledges them as essential signaling molecules with various, and often pleiotropic, functions (Bochkov et al., 2017). Researchers are investigating these molecules through untargeted approaches (Gil de la Fuente et al., 2018), targeted methods (Solati et al., 2021), or highly sophisticated mechanistic and functional studies (Slatter et al., 2018). Moreover, over the last decade, multiple highly advanced computational solutions were developed for redox lipidomics, with a particular focus on the robust identification of epilipids (Ni et al., 2019).

oxPCs are associated with cardiovascular diseases (Stamenkovic et al., 2017; Paynter et al., 2018), neurodegenerative diseases (Tyurina et al., 2015; Okuzumi et al., 2019), diabetes (Chen et al., 2018; Godzien et al., 2019; Nie et al., 2019), cancer (Reuter et al., 2010; Mantovani et al., 2012; Ingram et al., 2021), and non-alcoholic steatohepatitis (Ikura et al., 2006), all of which can be linked to oxidative stress and chronic inflammation. As reported previously, oxPCs may induce either beneficial or detrimental effects in the lungs (Ke et al., 2019; Karki and Birukov, 2020). Their impact may depend upon their structure and concentration; LCh-oxPCs exhibit a protective effect, and SCh-oxPCs are linked with disruptive effects. Moreover, lower levels of oxPCs protect the endothelial barrier, whereas high concentrations of the same species induce disruptive effects (Karki and Birukov, 2020). The beneficial effects of oxPCs are related to the protective effects on the endothelial barrier (through activation of Rac1, inhibition of Rho, activation of S1P1, or enhanced assembly of EC junctions) and anti-inflammatory effects (through inhibition of TLRs signaling, inhibition of NF-κB activation, increase in cAMP levels, activation of eNOS, Nrf2, HO-1 or LXA4 production) while detrimental effects are linked to the pro-inflammatory effects (through increased levels of MCP1, IL-6, IL-8, MIP-1, CXCL3, STAT3, NLRP3, TLR4, TLR2 CD36, and PRR activation), induction of coagulation (through increased expression of TF and decreased expression of TFPI and thrombomodulin), and increasing the endothelial permeability (via activation of Src kinase, ROS production, phosphorylation of VE-cadherin and EC junctional assembly disruption).

Inflammation and oxidative stress are heavily associated with two major health concerns worldwide: lung cancer and chronic obstructive pulmonary disease (COPD). Lung cancer is the most lethal type of cancer, taking 1.8 million lives annually (Sung et al., 2021). Non-small cell lung cancer (NSCLC) is the most prevalent type of lung cancer, accounting for 85%–90% of all cases (Duma et al., 2019). NSCLC is characterized by high malignancy, strong invasiveness, and easy metastasis, which, together with poor diagnosis and lack of effective treatment, result in high mortality. Therefore, early diagnosis, accurate clinical staging, and subtype determination are important to determine effective treatment plans, prolong survival, and improve the quality of life.

COPD and lung cancer are caused by cigarette smoking, and there is increasing evidence linking the two diseases beyond a common etiology. Smokers displaying airflow obstruction face a considerably elevated risk of lung cancer, with up to a fivefold increase compared to individuals with normal lung function. As pointed out by Durham and Adcock, the high prevalence of lung cancer in COPD suggests that there may be common mechanisms, such as inflammation, oxidative stress, premature aging in the lungs, genetic predispositions to either disease or common pathogenic factors, such as growth factors, activation of intracellular pathways or epigenetics (Durham and Adcock, 2015). Therefore, trying to obtain the oxPCs signature specific to lung cancer, as opposed to inflammation- and oxidative-stress-derived lung diseases, we decided to use COPD patients as our control group instead of healthy volunteers.

For many years, carcinogenesis and cancer proliferation have been mainly linked to disruptions in carbohydrate metabolism (Martinez-Outschoorn et al., 2017). However, over the last decade, researchers have revealed alterations in lipid metabolism and profound lipid reprogramming in malignant cells (Beloribi-Djefaflia et al., 2016; Gong et al., 2022).

Elevated oxidative stress has been reported in patients with lung cancer, but the exact mechanisms underlying this association are not fully understood (Mousapasandi et al., 2021). Previous reports have suggested that enhanced oxidative stress in patients with NSCLC occurs through either elevated formation of oxidation products or inactivation of antioxidant mechanisms (Sunnetcioglu et al., 2016). The overall redox status of NSCLC patients has already been investigated by other groups. However, in majority of cases, these studies have focused on end oxidation products, such as reactive aldehydes (Gęgotek et al., 2016; Karki and Birukov, 2020). Given the increased oxidative stress and elevated levels of phospholipids observed in NSCLC patients, it is reasonable to expect elevated levels of oxPCs. Moreover, the structure-related properties of different oxPCs highlight the importance of exploring distinct oxPC species. Therefore, the aim of this study was to determine the levels of oxPCs in NSCLC patients, including those with adenocarcinoma (ADC) and squamous cell carcinoma (SCC), the two most prevalent NSCLC subtypes, and investigate their potential role in the development of lung cancer.

## 2 Materials and methods

### 2.1 Chemicals and reagents

Ultrapure water was used to prepare all aqueous solutions and was obtained using a Milli-Q Integral 3 system (MilliporeSigma, Burlington, MA, United States). Zomepirac sodium salt, formic acid, LC-MS-grade methanol, acetonitrile, and LC-grade ethanol were purchased from Sigma-Aldrich (St. Louis, MO, United States).

### 2.2 Cohort

**FIGURE 1:**
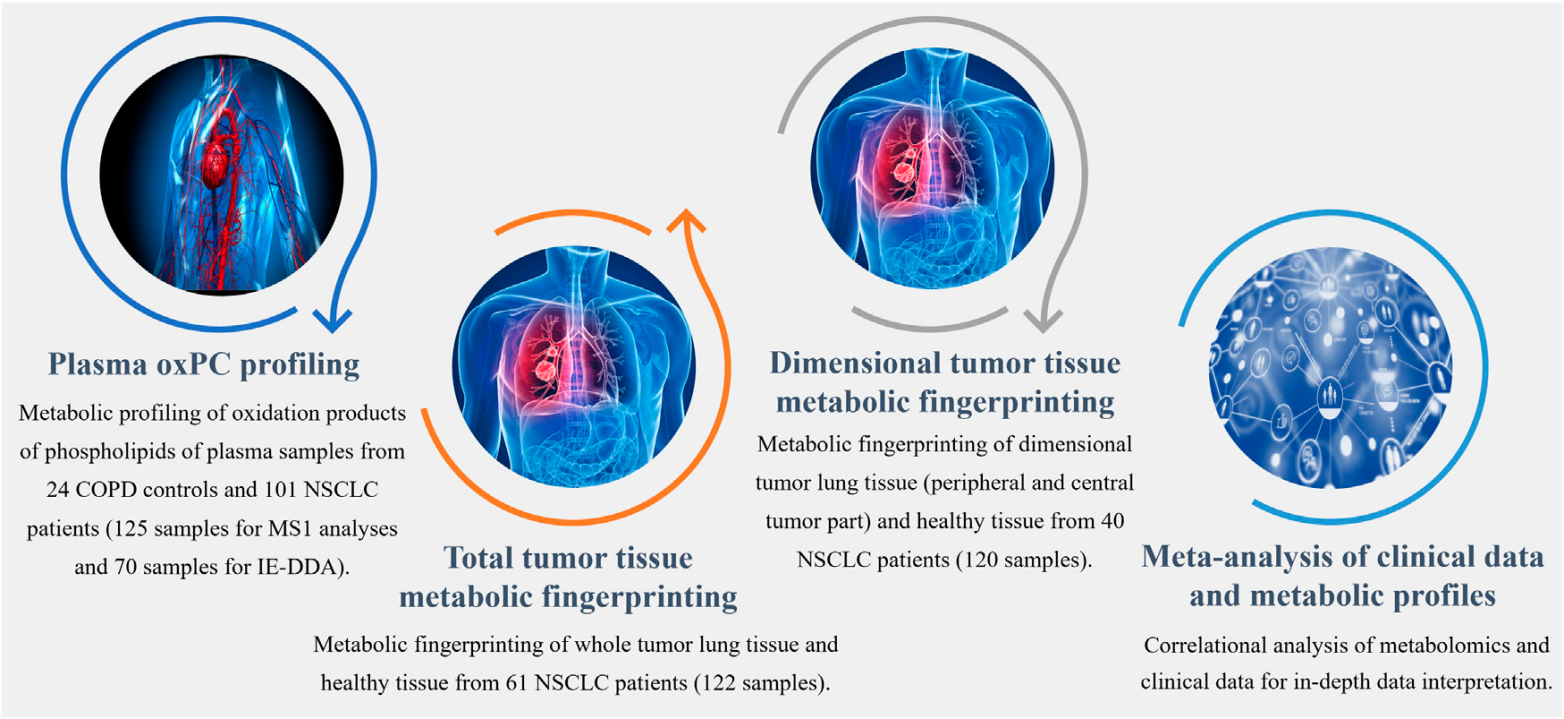
Workflow summarizing metabolomics analyses performed to explore the differential profiles of the oxPCs in COPD controls and NSCLC patients.

The study was approved by the Ethics Committee of the Medical University of Bialystok (R-I-003/262/2004, R-I-002/296/2018, and APK 002 5 2021) and was performed in accordance with the Declaration of Helsinki. Before sample collection, written informed consent for specimen collection was obtained from all participants. Samples were obtained from patients undergoing surgical treatment for primary NSCLC at the Department of Thoracic Surgery of the Clinical Hospital of the Medical University of Bialystok in Bialystok in Poland. Three distinct cohorts of patients were included (Figure 1), yielding 125 plasma and 242 tissue samples. Plasma samples were collected from 101 patients with NSCLC (41 ADC and 60 SCC patients) and 24 COPD controls. Control COPD group consisted of patients with an increased risk of NSCLC lung cancer diagnosed with chronic lung disease, chronic cough, wheezing or shortness of breath showing no focal lesions in lungs X-ray.

We collected tumor tissue and adjacent non-malignant control tissue from 61 patients with NSCLC (25 ADC and 36 SCC) for total tumor tissue metabolic fingerprinting, obtaining 122 samples. For dimensional tumor tissue metabolic fingerprinting, three pieces of tissues from central and peripheral tumor regions and adjacent non-malignant control tissues were collected from 40 NSCLC patients (20 ADC and 20 SCC) resulting in 120 samples. Clinicopathological characteristics, such as age, sex, smoking status, body mass index (BMI), tumor grade, lymph node metastases, histological type, clinical stage, and survival data were available. The basic clinical parameters describing each of the three cohorts enrolled in this study are summarized in Table 1.

**TABLE 1:** Basic clinical parameters of patients in three cohorts.

Plasma samples: oxPC profiling				
Patient characteristics	NSCLC (ADC + SCC) *n* = 101	ADC *n* = 41	SCC *n* = 60	COPD controls *n* = 24
Age [years] median (Q1–Q3)	63.0 (58.0–69.0)	62.0 (58.0–68.0)	63.5 (58.8–69.0)	63.0 (52.8–69.0)
BMI median (Q1–Q3)	25.47 (23.62–27.76)	25.99 (24.17–28.09)	25.39 (23.39–27.23)	24.56 (21.78–27.82)
Gender [F/M]	29/72	15/26	14/46	10/14
pTNM stage				
	*n* (%)	*n* (%)	*n* (%)	
IA	16 (16%)	10 (24%)	6 (10%)	
IB	10 (10%)	6 (15%)	4 (7%)	
IIA	24 (24%)	8 (20%)	16 (27%)	
IIB	33 (33%)	9 (22%)	24 (40%)	
IIIA	18 (18%)	8 (20%)	10 (17%)	
Tissue samples: total tumor tissue metabolic fingerprinting				
Patient characteristics	NSCLC (ADC + SCC) *n* = 61	ADC *n* = 25	SCC *n* = 36	
Age [years] median (Q1–Q3)	63.0 (56.0–69.0)	62.0 (55.0–69.0)	63.5 (57.5–69.0)	
BMI Median (Q1–Q3)	24.93 (23.41–26.09)	25.00 (23.53–26.37)	24.53 (23.39–25.39)	
Gender [F/M]	13/48	6/19	7/29	
pTNM stage				
	*n* (%)	*n* (%)	*n* (%)	
IA	0 (0%)	0 (0%)	0 (0%)	
IB	0 (0%)	0 (0%)	0 (0%)	
IIA	21 (34%)	9 (36%)	12 (33%)	
IIB	20 (33%)	8 (32%)	12 (33%)	
IIIA	20 (33%)	8 (32%)	12 (33%)	
Tissue samples: dimensional tumor tissue metabolic fingerprinting				
Patient characteristics	NSCLC (ADC + SCC) *n* = 40	ADC *n* = 20	SCC *n* = 20	
Age [years] median (Q1–Q3)	65.5 (59.0–69.0)	67.5 (59.8–69.5)	63 (59.0–69.0)	
BMI median (Q1–Q3)	26.565 (22.51–28.23)	27.04 (23.27–29.38)	26.255 (22.36–28.02)	
Gender [F/M]	20/20	10/10	10/10	
pTNM stage				
	*n* (%)	*n* (%)	*n* (%)	
IA	1 (3%)	1 (5%)	0 (0%)	
IB	10 (27%)	6 (30%)	4 (24%)	
IIA	2 (5%)	0 (0%)	2 (12%)	
IIB	9 (24%)	3 (15%)	6 (35%)	
IIIA	15 (41%)	10 (50%)	5 (29%)	

Whole blood was collected in 9 mL vacuum system tubes with K_2_EDTA as an anticoagulant. After gentle mixing, the plasma was separated by centrifugation at 1,300 × *g* for 20 min at room temperature. Plasma fractions (0.5 mL each) were collected in Eppendorf tubes and stored at −80°C until analysis.

Tissue samples were histologically reviewed and classified. After lung tumor resection, the whole specimen was examined macroscopically by a pathologist following previously published methods (Ciereszko et al., 2022) to determine the exact tumor localization, identify macroscopic tumor residuals, search for macroscopic infiltration of pulmonary pleura, and evaluate necrosis in the tumor center. The pathologist cut the tissue samples into tumor center and periphery subsamples. Moreover, the pathologist determined the possibility of collecting adjacent pulmonary tissue (referred to here as normal tissue); if the distance from the tumor border was greater than 2 cm, the pathologist took samples of adjacent tissue. Next, nurses from the biobank placed the tissue samples alternately into cryo-tubes containing the vapor phase of liquid nitrogen (fresh frozen samples) and into tubes with 10% buffered formalin (formalin-fixed samples). In this study we used fresh frozen samples.

Cancer stages were determined according to pathological tumor-node-metastasis (pTNM) staging. All tissue samples were frozen and stored at −80°C until analysis. Sample collection, quenching, and storage were performed according to the approved biobanking standards (Niklinski et al., 2017).

### 2.3 Clinical parameters

Serum C-reactive protein (CRP) and white blood cell (WBC) levels were determined the day before the surgical operation for tumor removal, together with other canonical biochemical parameters, in the diagnostic laboratory at the Clinical Hospital of the Medical University of Bialystok.

Metabolic tumor volume (MTV) and standardized uptake volume (SUV) were determined using simultaneous positron emission tomography-magnetic resonance imaging (PET/MRI) examinations performed using a Biograph mMR scanner (Siemens, Munich, Germany). Whole-body acquisition (from the top of the head to the mid-thigh) was begun 60 ± 10 min after the intravenous administration of 18-F-fluorodeoxyglucose at an activity of 4 MBq/kg body weight. Whole-body MRI was performed with T1- and T2-weighted images taken in the transverse plane; PET/MRI of the chest was performed using T1_vibe, T1_vibe_fatsat, T2_haste sequences, including breath-hold images were taken in the transverse, coronal, and sagittal planes and a magnetic resonance contrast agent was used in the absence of contraindications. The images were assessed by a nuclear medicine specialist and a radiologist with at least 5 years of experience. Radiotracer biodistribution was visually assessed, and semi-quantitative analysis was carried out using the metabolic activity index of lesions (SUVmax/lbm- SUV/lean body mass), and MTV was measured using a threshold-based method with 40% SUVmax.

### 2.4 Plasma and tissue sample preparation

Plasma samples were prepared using a previously described method (Daniluk et al., 2019). On the day of analysis, the samples were thawed on ice. For protein precipitation and metabolite extraction, one volume of plasma sample was mixed with three volumes of ice-cold methanol/ethanol (1:1) containing 1 ppm of zomepirac, used as an internal standard (IS). After extraction, the samples were stored on ice for 10 min and centrifuged at 21,000 × *g* for 20 min at 4°C. The supernatant was filtered through a 0.22 μm nylon filter (Thermo Fisher Scientific, Waltham, MA, United States).

Tissue samples were prepared according to a previously described method (Ciborowski et al., 2017). On the day of analysis, the samples were thawed on ice. Ten milligrams of lung tissue were placed in an Eppendorf tube with two stainless steel beads (5 mm) and 200 μL of ice-cold 50% methanol. Samples were homogenized for 8 min at 30 Hz using a TissueLyser LT instrument (Qiagen, Hilden, Germany). After homogenization, the beads were removed, and 200 μL of ice-cold acetonitrile containing 1 ppm of zomepirac (internal standard) was added to the sample. Metabolites were extracted by vortexing the samples for 1 h. After extraction, the samples were centrifuged at 21,000 × *g* for 20 min at 20°C. The supernatant was filtered through a 0.22 μm nylon filter. The extraction blank was prepared following the same procedure as the biological samples but without tissue, and was analyzed together with biological samples.

Quality control samples (QCs) were prepared by mixing equal volumes of raw plasma and equal volumes of metabolite extract of tissue samples. QCs were treated like the rest of the samples and injected at the beginning of the batch (10 injections) to equilibrate the system and after every ten samples to monitor the stability of the measurement (Godzien et al., 2014).

### 2.5 Analytical setup

Three types of analysis were performed, covering plasma oxPC profiling, total tumor tissue metabolic fingerprinting and dimensional tumor tissue metabolic fingerprinting. However, recoded data was processed in a targeted manner, retrieving from it solely information about oxPCs.

Plasma oxPC profiling was performed using a 6546 iFunnel ESI-QTOF (Agilent Technologies, Santa Clara, CA, United States) coupled with a 1290 Infinity UHPLC system (Agilent Technologies) with a degasser, quaternary pump, and thermostatted autosampler.

Tissue metabolic fingerprinting was performed using a 6545 iFunnel ESI-QTOF instrument (Agilent Technologies, Santa Clara, CA, United States) coupled with the 1290 Infinity UHPLC system (Agilent Technologies) with a degasser, binary pump, and thermostatted autosampler.

Plasma and tissue samples were analyzed in both polarity modes. During all analyses, two reference compounds were used: *m/z* 121.0509 (protonated purine) and *m/z* 922.0098 (protonated hexakis (1H,1H,3H-tetrafluoropropoxy)phosphazine [HP-921]) for the positive ionization mode, and *m/z* 112.9856 (proton abstracted trifluoroacetic acid anion) and *m/z* 966.0007 (formate adduct of HP-921) for the negative ionization mode. These masses were continuously infused into the system to allow internal constant mass correction during data acquisition.

All datasets were acquired in both polarity modes. However, data analysis revealed that more abundant and, therefore, more reproducible signals of oxPCs were obtained in the positive ion mode. The negative-ion mode provided more detailed structural information. Consequently, information from the positive ion mode was used for statistical analyses, whereas information acquired in the negative ion mode was used for lipid annotation.

### 2.6 Plasma oxPC profiling

Four microliters of each sample were injected into a thermostatted Zorbax Extend C18 column (RRHT 2.1 × 50 mm, 1.8 μm; Agilent Technologies) at 60°C. The flow rate was 0.6 mL/min for aqueous phase A (water with 0.1% formic acid) and organic phase B (acetonitrile with 0.1% formic acid). The chromatographic gradient started at 50% phase B, then increased to 80% (1–6 min) and 100% (6–8 min). Finally, the system was re-equilibrated by reverting the phase composition to initial conditions (50% phase B) in 0.5 min, and this was maintained from 8.5 to 10 min. The mass spectrometer was operated in full-scan mode (MS1). Data were acquired at *m/z* values ranging from 50 to 1,000 at a scan rate of 1.0 scan per second. The drying gas flow rate was 12 L/min, the temperature was 250°C, and the gas nebulizer pressure was set to 52 psig. The nozzle voltage was 1,000 V, and the capillary voltages were 3,000 and 4,000 V in the positive and negative ion modes, respectively.

All samples were analyzed in scan mode (MS1) for both polarities. Then, a subset of 70 samples was analyzed in the negative ion mode using iterative exclusion data-dependent analysis (IE-DDA). The precursor ions were fragmented using ramped collision energy adjusted for each molecule according to its *m/z* value. The first injection was performed as a conventional data-dependent analysis where the top three most abundant precursors were selected for fragmentation considering the active exclusion lists. During the subsequent injection, precursors selected for MS/MS fragmentation in the previous injection were excluded on a rolling basis with a mass error tolerance and 0.5 min retention time tolerance. Five iterative MS/MS runs were performed for each sample, resulting in 350 measurements.

### 2.7 Lung tissue metabolic fingerprinting

One microliter of the extracted sample was injected into a Zorbax Eclipse Plus C8 column (RRHD 2 × 150 mm, 1.8 μm; Agilent Technologies) at 60°C. The flow rate was 0.6 mL/min for aqueous phase A (water with 0.1% formic acid) and organic phase B (acetonitrile with 0.1% formic acid). The gradient started at 25% phase B and increased to 95% phase B over 14 min. This was maintained for 1 min, and then the gradient returned to starting conditions (25% phase B) in 0.1 min and maintained for 4.9 min to re-equilibrate the system before the next injection. The mass spectrometer was operated in the full-scan mode (MS1) from *m/z* 50 to 1,000. The drying gas flow rate was 12 L/min, the temperature was 250°C, and the gas nebulizer pressure was set to 52 psig. The nozzle voltage was 1,000 V, and the capillary voltages were 3,000 and 4,000 V in the positive and negative ion modes, respectively.

### 2.8 Determination of protein content in lung tissue

The precipitated proteins were suspended in radioimmunoprecipitation assay (RIPA) buffer, denatured at 60°C, and sonicated for 30 min in a water bath. The samples were then centrifuged for 15 min at 14,000 × *g*. Protein concentration was measured using the Pierce BCA Protein Assay Kit (Thermo Fisher Scientific) according to the manufacturer’s protocol.

### 2.9 Data processing: plasma oxPC profiling

MS1 data were reprocessed using a targeted approach to extract from the acquired data information about oxPCs (Godzien et al., 2019). IE-DDA data were used to confirm the annotation of oxPCs based on the MS/MS spectra. For this purpose, we searched for known fragmentation patterns, inspecting acquired MS/MS spectra in Mass Hunter Qualitative software (Agilent Technologies, B.07.00) (Gil de la Fuente et al., 2018). Moreover, all annotations were confirmed using retention time to compare the elution order between different oxPCs and their non-oxidized precursors. This step was crucial for minimizing false annotations of in-source generated lipids. A list of 45 oxPCs was defined (Supplementary Table S1) and covered 13 LCh-oxPCs and 32 SCh-oxPCs (including isoforms). OxPCs for which we found isomeric forms are denoted with the postfix “iso” followed by the number, signifying the identification of multiple isomers. Conversely, oxPCs lacking this “iso” prefix indicate that isoforms were not detected for those specific compounds.

Retention time and mass data pairs of annotated oxPCs were used as input criteria to search them in MS1 data. This processing was performed using the “Find by Ion” algorithm in Mass Hunter Profinder software (Agilent Technologies, B.10.00). The integration of all extracted peaks was manually curated and corrected if necessary.

### 2.10 Data processing: tissue metabolic fingerprinting

MS1 data were reprocessed using a targeted approach to extract information about oxPCs from the acquired data (Godzien et al., 2019). A list of 45 oxPCs was defined (Supplementary Table S1) and covered 13 LCh-oxPCs and 32 SCh-oxPCs (including isoforms). Annotation of these oxPCs was done in previous projects based on the data-independent analysis (DIA), and incorporated into in-house built library. Retention time and mass data pairs of annotated oxPCs were used as input criteria to search them in MS1 data. This processing was performed using the “Find by Ion” algorithm in Mass Hunter Profinder software (Agilent Technologies, B.10.00). The integration of all extracted peaks was manually curated and corrected if necessary.

### 2.11 Statistical analysis

The acquired data underwent evaluation through a Quality Assurance procedure. oxPCs displaying a Relative Standard Deviation (RSD) of signals in QC samples below 30% were deemed reliably measured and retained for subsequent statistical analyses.

Before statistical analyses, data from the plasma analyses were normalized solely to the IS to minimize analytical drift. Data from the tissue analyses were normalized to the IS and to protein content to minimize differences between different pieces of tissue. Before using IS data for normalization purposes, the quality of measured signals and integrated peaks was evaluated, checking the RSD of IS across all the samples to ensure that its signal was not interfered by co-eluting molecules changing between compared groups.

Plasma samples were analyzed for general comparisons of *i*) control vs*.* NSCLC; for subtype comparison of *ii*) control vs*.* ADC subtype and *iii*) control vs*.* SCC subtype. Tissue samples were analyzed for general comparison between *i*) tumor and non-cancerous lung tissue of NSCLC; for subtype comparison between *ii*) tumor and non-cancerous lung tissue in ADC and *iii*) tumor and non-cancerous lung tissue in SCC.

Differences between compared groups were described with *p*-values and relative differences expressed as percentages, where a positive value indicated higher values in cancer patients than in controls, and a negative value indicated lower signals in cancer patients than in controls. *p*-values were computed using a non-parametric Mann-Whitney test in Mass Profiler Professional (MPP, Agilent Technologies 15.1). For plasma comparisons between NSCLC and COPD patients unpaired test was applied, while for the tissue comparisons of different tissue sections from the same patients paired test was employed. Obtained *p*-values were corrected employing Benjamini–Hochberg False Discovery Rate correction. However, because of small number of tested variables uncorrected *p* < 0.05 was considered significant.

Variations between groups were inspected by calculating the percentage of change in Excel (Microsoft) and fold change in MPP 15.1 (Agilent Technologies). Furthermore, we calculated using Excel (Microsoft) the median, Q1, and Q3 for each oxPC level in each comparison (Tables 2, 3). To evaluate the prevalence of elevated oxPC levels, we determined the percentage of patients in whom increased levels of oxPCs were observed in tumor tissue compared to healthy lung tissue.

**TABLE 2:** Differences in oxPC profiles of NSCLC patients and COPD controls in plasma samples (Panel A) and non-cancerous lung tissue and tumor tissue from NSCLC patients (Panel B).

A) Relative plasma oxPC abundance between NSCLC patients and COPD controls					
oxPC	Raw *p*-value (Corrected *p*-value)	Change [%] (Fold change)	COPD controls	NSCLC	
			Median abundance (Q1–Q3) [counts]		
PC 16:0/20:3; OH	2.46E-14	NSCLC^a^	0 (0–0)	87,985 (76,390–99,205)	
	(3.20E-13)	(16.00)			
PC 16:0/18:2; OH	1.95E-13	179	62,930 (56,807–72,151)	145,245 (117,414–190,046)	
	(1.26E-12)	(2.46)			
PC 16:0/20:4; OH	2.36E-11	88	107,090 (89,664–114,582)	164,852 (139,989–204,070)	
	(1.02E-10)	(1.74)			
PC 18:0/20:4; OH	3.23E-08	54	113,525 (106,043–130,812)	154,079 (131,649–201,613)	
	(1.05E-07)	(1.44)			
PC 18:0/18:2; OH	2.44E-02	17	216,912 (179,508–247,975)	232,874 (207,020–285,790)	
	(5.29E-02)	(1.15)			
PC 18:0/20:4; OOH	1.75E-02	14	92,188 (84,837–101,544)	102,019 (90,049–117,132)	
	(4.55E-02)	(1.13)			

B) Relative oxPC levels between healthy lung tissue and tumor tissue from NSCLC patients					
oxPC	Raw *p*-value (Corrected *p*-value)	Change [%] (Fold change)	Healthy tissue	Tumor tissue	
			Median abundance (Q1–Q3) [counts]		
PC 16:0/7:0; COOH iso4	6.61E-05	87	920 (646–1,368)	1,440 (648–2,761)	
	(6.61E-05)	(1.70)			
PC 16:0/7:0; COOH iso2	8.66E-05	10	290 (199–568)	367 (214–644)	
	(6.06E-04)	(1.09)			
PC 16:0/5:0; COOH	2.05E-02	92	567 (433–868)	458 (0–663)	
	(4.79E-02)	(1.34)			
PC 16:0/5:0; CHO	7.79E-03	73	1,322 (821–1,867)	1,561 (1,089–2,640)	
	(2.18E-02)	(1.30)			
PC 16:0/4:0; CHO iso2	5.62E-03	65	539 (285–754)	636 (356–899)	
	(1.97E-02)	(1.24)			
PC 16:0/18:2; OH iso2	1.59E-03	76	3,622 (2,954–4,592)	4,832 (3,829–6,452)	
	(7.44E-03)	(1.37)			

Raw *p*-value computed with the Mann-Whitney unpaired test for plasma comparisons and paired test for tissue comparisons. Corrected *p*-value was calculated employing Benjamini–Hochberg FDR, correction.

The positive value of the change indicates that the average amount of oxPC, is higher in plasma of cancer patients than in controls, and higher in tumor tissue than in healthy lung tissue, while a negative value indicates that the average amount of oxPC, is lower in plasma of cancer patients than in controls, and lower in tumor tissue than in healthy lung tissue.

^a^ NSCLC, oxPCs, observed only in NSCLC, samples and not in COPD.

**TABLE 3:** Differential oxPC profiles between ADC, SCC, and COPD patients in plasma (Panel A) and lung tissue (Panel B).

A) oxPC levels between ADC and SCC patients and COPD controls in plasma							
oxPC	ADC		SCC		COPD controls	ADC	SCC
	Raw *p*-value (Corrected *p*-value)	Change [%] and Fold change	Raw *p*-value (Corrected *p*-value)	Change [%] and Fold change	Median abundance (Q1–Q3) [counts]		
PC 16:0/20:3; OH	6.77E-12	ADC	5.45E-13	SCC	0 (0–97,285)	89,546 (80,989–97,285)	81,998 (73,048–102,407)
	(8.79E-11)	(16.00)	(7.09E-12)	(16.00)			
PC 16:0/18:2; OH	5.19E-11	220	8.37E-12	150	62,930 (56,807–197,594)	166,556 (133,276–197,594)	126,730 (108,060–171,867)
	(3.37E-10)	(2.77)	(5.44E-11)	(2.27)			
PC 16:0/20:4; OH	7.38E-10	99	1.28E-09	80	107,090 (89,664–194,518)	172,742 (151,500–194,518)	161,365 (131,994–207,264)
	(3.20E-09)	(1.81)	(5.56E-09)	(1.69)			
PC 18:0/20:4; OH	7.33E-08	72	1.49E-06	43	113,525 (106,043–212,391)	170,713 (141,629–212,391)	149,667 (129,091–196,851)
	(2.38E-07)	(1.55)	(4.85E-06)	(1.37)			
PC 18:0/18:2; OH	2.16E-02	23	ns	13	216,912 (179,508–289,560)	246,907 (212,977–289,560)	227,509 (206,884–280,635)
	(5.62E-02)	(1.19)		(1.12)			
PC 18:0/20:4; OOH	ns	10	8.95E-03	17	92,188 (84,837–118,456)	98,488 (87,384–118,456)	103,146 (92,983–116,214)
		(1.09)	(2.33E-02)	(1.15)			
PC 16:0/7:0; COOH	ns	6	2.16E-02	23	8,564 (6,614–12,101)	9,346 (6,642–12,101)	10,701 (8,059–14,467)
		(1.04)	(4.68E-02)	(1.22)			

B) oxPC levels between healthy lung tissue and tumor tissue from ADC and SCC patients								
oxPC	ADC		SCC		ADC healthy tissue	ADC tumor tissue	SCC healthy tissue	SCC tumor tissue
	Raw *p*-value (Corrected *p*-value)	Change [%] and Fold change	Raw *p*-value (Corrected *p*-value)	Change [%] and Fold change	Median abundance (Q1–Q3) [counts]			
PC 16:0/7:0; COOH iso4	5.76E-05	64	4.60E-02	104	888 (665–1,057)	1,779 (1,259–2,537)	1,046 (568–1,469)	1,146 (593–3,007)
	(8.06E-04)	(1.79)	(1.60E-01)	(1.65)				
PC 16:0/5:0; COOH	ns	38	3.02E-02	138	293 (224–643)	377 (214–662)	278 (183–559)	352 (219–616)
		(1.01)	(1.60E-01)	(1.65)				
PC 16:0/5:0; CHO	ns	22	4.96E-02	113	1,322 (750–2,259)	1,691 (1,167–2,640)	1,321 (843–1,810)	1,549 (907–2,548)
		(1.19)	(1.60E-01)	(1.38)				
PC 16:0/4:0; CHO iso2	4.80E-02	24	ns	96	561 (325–754)	612 (432–914)	515 (277–757)	673 (273–852)
	(2.02E-01)	(1.16)		(1.30)				
PC 16:0/18:2; OH iso2	5.45E-04	93	ns	66	3,658 (3,304–4,237)	5,399 (4,247–6,436)	3,413 (2,828–5,150)	4,349 (2,697–6,645)
	(3.82E-03)	(1.62)		(1.21)				

Raw *p*-value computed with the Mann-Whitney unpaired test for plasma comparisons and paired test for tissue comparisons. Corrected *p*-value was calculated employing Benjamini–Hochberg FDR, correction.

The positive value of the change indicates that the average amount of oxPC, is higher in plasma of cancer patients than in controls, and higher in tumor tissue than in healthy lung tissue, while a negative value indicates that the average amount of oxPC, is lower in plasma of cancer patients than in controls, and lower in tumor tissue than in healthy lung tissue.

ns–non-significant.

Metabolomic data were correlated with complex clinical data, including WBC, CRP, PET/MRI, MTV, and SUV values. Spearman correlation analysis was performed using MetaboAnalyst 5.0 (Pang et al., 2021).

## 3 Results

### 3.1 Baseline characteristics and general concerns

The baseline characteristics of the patients with NSCLC and COPD controls are summarized in Table 1. The enrolled subjects were matched without any statistical differences between groups in basic anthropometric measurements. However, more male than female participants were enrolled, reflecting the higher incidence of lung cancer in men compared to women during the sampling period (Mederos et al., 2020). The number of samples per group, splitting the cohort according to the gender, was too small to perform robust correlational analysis, therefore to check if there are gender specific differences in the oxPCs level, we calculated the percentage of change between compared groups separately for female and male. Results of this analysis are summarized in Supplementary Table S2.

### 3.2 Annotation of oxPCs in plasma

Plasma analyses were performed on samples collected from 101 NSCLC patients and 24 COPD controls. Supervised data processing resulted in the annotation of 13 of the 45 oxPCs used for the target search (Supplementary Table S1). These oxPCs were annotated based on DDA-MS/MS structural information and fragmentation rules (Gil de la Fuente et al., 2018; Lange et al., 2021). Among the oxPCs identified, five were SCh-oxPCs and eight were LCh-oxPCs. Among the LCh-oxPCs, six contained hydroxy groups and only two contained hydroperoxy groups. We found three aldehyde- and two carboxylic acid-containing SCh-oxPCs. After the QA procedure, we rejected four oxPCs (one LCh-oxPC and three SCh-oxPC) which RSD was above 30%. For all other oxPCs RSD for QC samples was below 30% with an average of 21.4%.

### 3.3 Annotation of oxPCs in tumor tissue

Total tumor analyses were performed on 122 samples collected from 61 patients with NSCLC: two samples were collected from each patient, covering healthy tissue and tumor tissue. Supervised data processing allowed for the annotation of 16 out of the 45 oxPCs used for the target search (Supplementary Table S1). These oxPCs were annotated based on exact mass and RT matching with the in-house built library. Among these, 15 were SCh-oxPCs and only one was an LCh-oxPC. The LCh-oxPC contained a hydroxy group. Among the SCh-oxPCs, we found nine aldehyde- and six carboxylic acid-containing forms. After the QA procedure, we rejected two oxPCs (one LCh-oxPC and one SCh-oxPC) which RSD was above 30%. For all other oxPCs RSD for QC samples was below 30% with an average of 13.9%.

Dimensional tumor tissue analyses were performed on 120 samples collected from 40 NSCLC patients: three samples were collected from each patient, covering healthy tissue, peripheral tumor tissue and central tumor tissue. Supervised data processing resulted in the annotation of 20 out of the 45 oxPCs used for the target search (Supplementary Table S1). These oxPCs were annotated based on exact mass and RT matching with the in-house built library. Among these oxPCs, 14 were SCh-oxPCs and six were LCh-oxPCs. Among the LCh-oxPCs, three contained hydroxy groups and three contained hydroperoxy groups. Among the SCh-oxPCs, we found five aldehyde- and nine carboxylic acid-containing forms. After the QA procedure, we rejected five oxPCs (two LCh-oxPC and three SCh-oxPC) which RSD was above 30%. For all other oxPCs RSD for QC samples was below 30% with an average of 12.3%.

### 3.4 oxPCs in NSCLC vs*.* COPD: a general comparison

**FIGURE 2:**
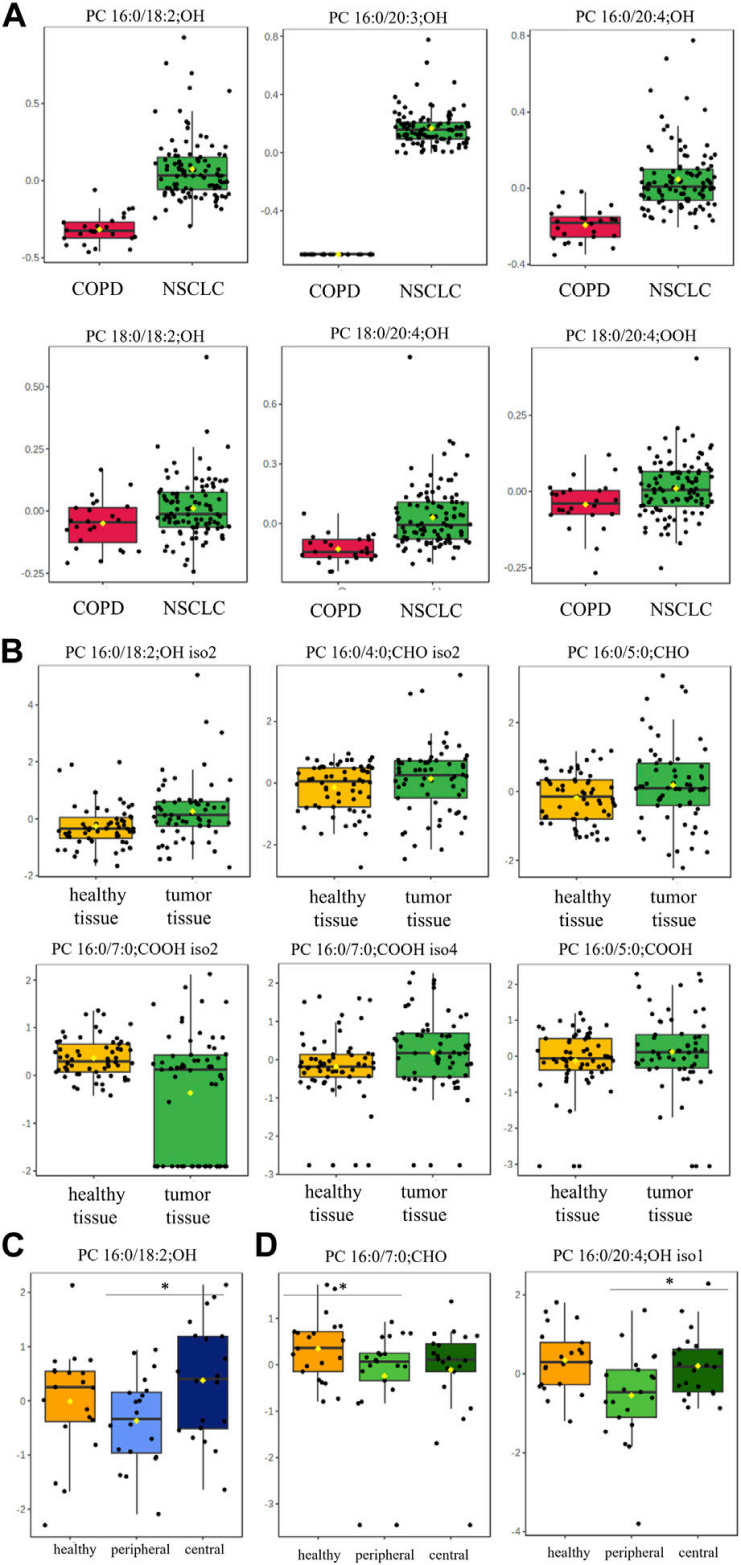
Box-and-whisker plots comparing oxPC levels between 101 NSCLC patients and 24 COPD controls in plasma samples (Panel **(A)**) and between healthy lung tissue and tumor tissue from 61 NSCLC patients (Panel **(B)**); non-malignant tissue and in peripheral and central tumor regions in 20 ADC patients (Panel **(C)**) and 20 SCC patients (Panel **(D)**). The levels of oxPCs shown on the figure were found to be significantly different between the compared groups according to the Mann-Whitney unpaired test for plasma comparisons and paired test for tissue comparisons. The y-axis in the graph represents the normalized signal of oxPCs (counts), which have been log-transformed and UV-scaled. The black dots in the graph represent the concentrations of oxPCs from all samples in a given group. The notch represents the 95% confidence interval around the median of each group, which is defined as ±1.58 times the interquartile range divided by the square root of the number of samples. The mean concentration of each group is indicated with a yellow diamond in the graph.

oxPC levels were significantly higher in NSCLC patients than in COPD patients (Table 2). The variation among samples in the NSCLC group was greater than that in the COPD group (Figure 2A). An overall general increase in oxPC levels was also observed in tissue samples; in NSCLC patients, tumor tissues exhibited higher levels of oxPCs than healthy lung tissue (Figure 2B). The magnitude of this difference was smaller than in plasma samples. We also inspected the levels of oxPCs in each patient separately, comparing the levels of oxPCs between healthy and tumor tissues, and we observed elevated oxPC levels in tumor tissue in 63% of NSCLC patients.

Dimensional tissue analyses revealed that oxPC levels were overall lower in the peripheral tumor regions in comparison to both, healthy tissue and central tumor region (Figures 2C, D).

### 3.5 oxPCs in NSCLC vs*.* COPD: NSCLC subtypes

**FIGURE 3:**
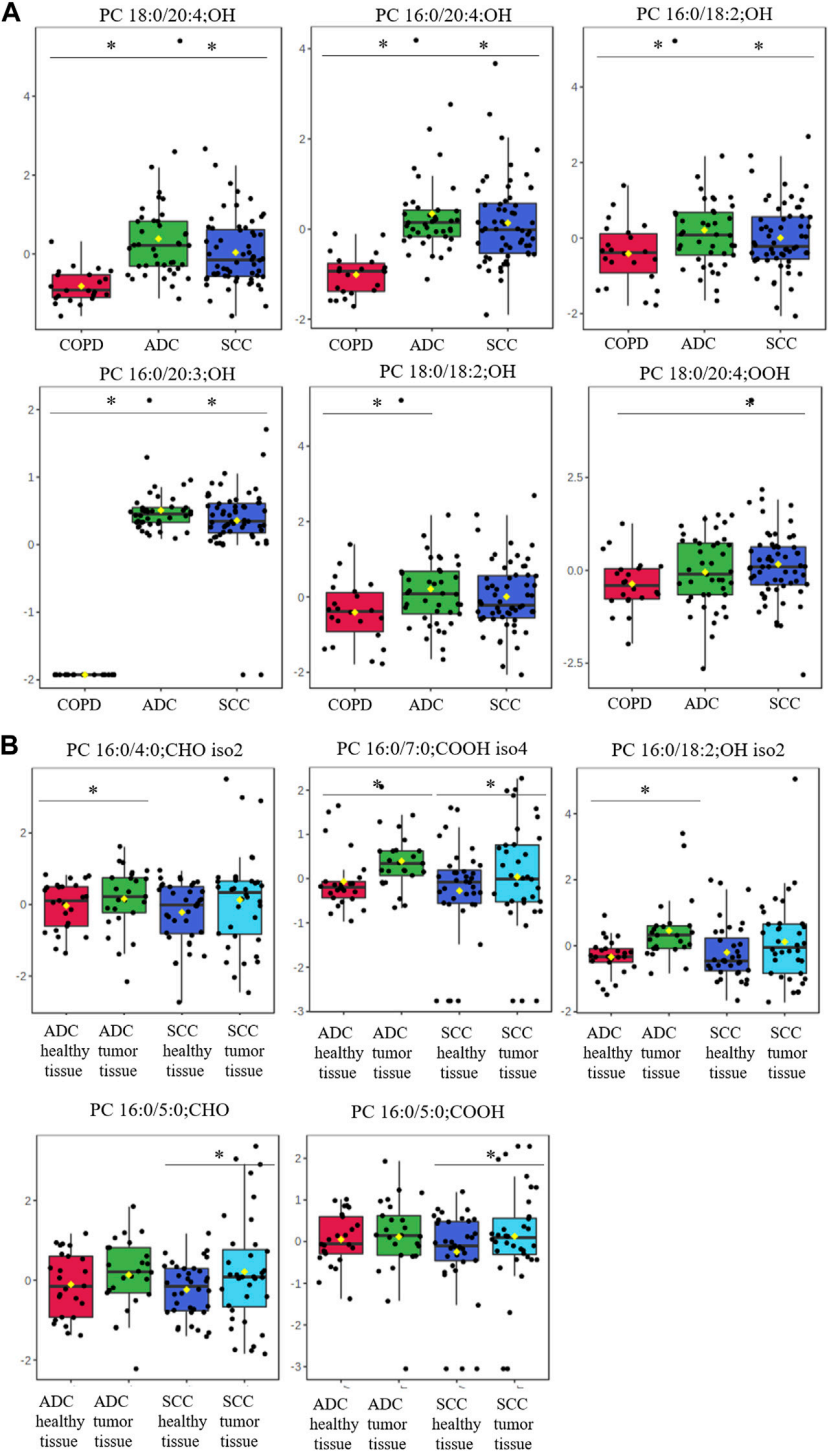
Box-and-whisker plots comparing oxPCs levels between plasma samples from 41 ADC and 60 SCC patients and 24 COPD controls (panel **(A)**) and between healthy, non-malignant tissue and tumor tissue in 25 ADC and 36 SCC patients (panel **(B)**). The levels of oxPCs shown on the figure were found to be significantly different between the compared groups according to the Mann-Whitney test. The y-axis in the graph represents the normalized signal of oxPCs (counts), which have been log-transformed and UV-scaled. The black dots in the graph represent the concentrations of oxPCs from all samples in a given group. The notch represents the 95% confidence interval around the median of each group, which is defined as ±1.58 times the interquartile range divided by the square root of the number of samples. The mean concentration of each group is indicated with a yellow diamond in the graph. * indicates statistically significant comparisons.

Next, we assessed oxPC levels by NSCLC subtype (ADC and SCC). We observed a generally higher elevation of oxPC levels in the plasma and tumor tissues of ADC patients than in SCC patients (Figure 3). The observed differences were larger for plasma than for tissue samples. In addition, the magnitude of the difference in oxPC levels between COPD and ADC patients was higher than that between COPD and SCC patients (Table 3). Similar findings were also observed in tissue samples, where a greater difference in oxPC levels was observed between tumor and healthy lung tissue in patients with ADC compared to patients with SCC. Comparisons of tissue oxPC levels, between healthy and tumor tissue, for individual patients revealed elevated levels in 85% of ADC patients and only 56% of SCC patients.

### 3.6 Correlation analysis

Correlation analysis between plasma oxPC levels and clinical parameters revealed a statistically significant (*p*-value<0.05) but weak (correlation coefficient less than 0.5) negative correlation between two oxPCs (PC 16:0/20:3; OH and PC 16:0/18:2; OH) and WBC. No statistically significant correlation was observed between oxPC and CRP levels.

Correlation analysis of tumor oxPC levels with metabolic activity index of lesions, expressed as SUV and MTV, did not reveal any correlations, regardless of whether the correlation was calculated for all NSCLC patients or for only ADC or SCC patients.

## 4 Discussion

The obtained oxPC profiles of the plasma and tumor tissue were different. LCh-oxPCs were the predominant form in the plasma, accounting for 62% of all detected oxPCs. In contrast, SCh-oxPCs were the primary fraction in tissues, representing 94% and 70% of all detected oxPCs in the total tumor and dimensional tissue analyses, respectively. Solati et al. (2021) reported a similar observation of locally predominant SCh-oxPCs in the thrombi of patients with ST-segment elevation myocardial infarction tissue, however it was a different tissue and disease context. It is important to emphasize that the results obtained from plasma samples pertain to the comparison between NSCLC and COPD patients. Conversely, the results obtained from tissue samples involve the comparison between cancerous and healthy lung tissues from NSCLC patients. Therefore, to compare these results directly, ideally, we should include also lung tissue from COPD patients, however we were not able to obtain such biopsy, which is one of the limitations of this study.

We observed an overall elevation of oxPCs in NSCLC patients, including elevated plasma oxPC levels compared to controls and elevated tissue oxPC levels in tumor tissue compared to healthy lung tissue (Figure 2). We observed an overall elevation of oxPC levels in patients with NSCLC compared to those in COPD controls. Considering that oxidative stress is a hallmark of numerous airway diseases (Sunnetcioglu et al., 2016), it can be assumed that the observed elevation would be even more significant if patients with NSCLC were compared with healthy controls.

An interesting observation was made for the results obtained considering gender differences. As can be seen in Supplementary Table S2, a higher magnitude of change is observed for males than for females. This phenomenon is attributed to the protective antioxidant properties of estrogen, frequently combined with lower exposure to exogenous risk factors, including alcohol and tobacco use, leading to diminished levels of ROS generation and mitochondrial impairment in females compared to males (Ethun, 2016; Allegra et al., 2023). However, it is important to mention, that according to the evidence, the number of smoking women during the last decade is increasing, resulting in a higher prevalence of NSCLC in women but also weakening their antioxidant defense capacity (Jafari et al., 2021).

Sunnetcioglu et al. (2016) investigated oxidative damage in three respiratory diseases (COPD, lung cancer, and obstructive sleep apnea syndrome) and revealed oxidative stress and stress responses in plasma samples for all three diagnoses. The highest level of oxidative stress, reflected via levels of coenzyme Q10 and 8-oxo7,8-dihydro-2-deoxyguanosine (8-oxodG), was observed in lung cancer. MDA levels were highest in patients with sleep apnea, but were also significantly higher in lung cancer and COPD patients than in controls. Primary oxidation products are known to be intermediates in the formation of end products. Therefore, it is crucial to investigate the relationship between them in the context of oxidative stress in NSCLC. For instance, higher levels of primary products may be associated with lower levels of end products. In comparison, lower levels of primary products could be indicative of a greater accumulation of end products. The reported elevation of end oxidation products and our observation of elevated levels of primary oxidation products suggest that oxidative stress is elevated to such an extent that all oxidation products are increased.

Although both types of oxPCs originate from the same precursors, their formation and biological activity differ. LCh-oxPCs can be formed enzymatically and non-enzymatically, whereas SCh-oxPCs are formed only non-enzymatically (Davies and Guo, 2014). Our data do not provide details about the origin of LCh-oxPCs in the plasma. Furthermore, the measured quantities may correspond to both enzymatically and non-enzymatically formed LCh-oxPCs. Therefore, precise interpretation of the obtained plasma results is challenging.

The origin of tissue SCh-oxPCs is non-enzymatic and most likely induced by ROS (Davies and Guo, 2014). Dimensional tissue analyses revealed that oxPC levels were lower in the peripheral than in the central region of the tumors and healthy lung tissue (Figures 2C, D). It can be concluded that the elevated level of oxPCs observed in tumor tissue resulted from the accumulation of these lipids in the central region. To the best of our knowledge, this is the first study to report the spatial localization of oxPCs in tumor tissue.

The elevated levels of oxPCs in healthy tissue might be linked to the smoking, since it induces oxidative stress. The formation and accumulation of SCh-oxPCs in tumor tissue may occur due to hypoxia of the metabolically hyperactive growing tumor, along with insufficient vascularization (Emami Nejad et al., 2021). Moreover, in agreement with our previous study (Kowalczyk et al., 2021), Marien et al. (2015) demonstrated changes in phospholipid profiles in NSCLC patients, indicating an increase in phosphatidylcholine species in tumor tissue compared to that in healthy lung tissue. Furthermore, previous studies have reported a reduced antioxidant capacity in malignant lung tissues (Gęgotek et al., 2016). Therefore, accumulated phosphatidylcholines can be easily oxidized under hypoxic conditions to form oxPCs. This may explain the accumulation of oxPCs in the tumor.

The magnitude of observed differences between groups in this study was generally smaller in tumor tissues than in the plasma samples (Figures 2, 3). This may be because plasma samples were collected from NSCLC patients and compared with samples from COPD patients, whereas tissue samples (tumor and non-cancerous tissue) were collected from NSCLC patients. Although histologically malignant and non-malignant tissues were separated, non-malignant tissues were collected from the surrounding area, and were likely affected by tumor metabolism. Moreover, tumors are complex and highly heterogeneous structures comprising many different cellular and noncellular elements (Xing et al., 2010). Therefore, different pieces of tumor tissue may not have exactly the same metabolic content, which might contribute to the observed intra-group heterogeneity reflected in the wide range of measured oxPC levels. This phenomenon is common for these molecules and has been observed previously (Solati et al., 2021).

We correlated the levels of tissue oxPCs with MTV values, which reflect tumor size and SUV, which provide evidence of tumor activity. We expected to observe a correlation between the MTV values and the levels of oxPCs since greater tumor size can result in poorer oxygenation leading to increased oxidative stress and consequently higher levels of oxPCs. Similarly, we expected to observe a correlation between SUV values and the levels of oxPCs since metabolic hyperactivity often leads to increased oxidative stress and subsequently higher levels of oxPCs. Surprisingly, no SCh-oxPCs showed a correlation with tumor size or activity. This finding may suggest the non-enzymatic origin of these oxidative products. Indeed, (ROS)-induced formation of oxPCs is uncontrolled and generates wide range oxidation products with wide range of concentrations (O'Donnell et al., 2019).

We observed some differences in oxPC levels between the two cancer subtypes and COPD controls. Our data showed a greater elevation of oxPC levels relative to controls in ADC patients than in SCC patients. This elevation was also more common in the tumor tissue of ADC patients (85%) than in that of SCC patients (56%). Differences between the two NSCLC subtypes concerning redox status have already been reported; however, to our knowledge, this is the first report regarding primary oxidation products. Gęgotek et al. (2016) reported alterations in tissue MDA and 4-HNE levels between ADC and SCC patients. MDA was nearly four times higher in SCC than in healthy tissue and six times higher in ADC than in healthy tissue. Differences in 4-HNE levels were not as substantial (2.7 and 2.3 times higher in SCC and ADC, respectively, than in healthy tissues). Elevated 4-HNE levels relative to those in healthy tissues were seen in 83% of ADC tissues and 46% of SCC tissues. Gęgotek et al. (2016) concluded that lipid peroxidation is more involved in ADC development, whereas endocannabinoids contribute more to SCC growth and development. While we cannot draw conclusions regarding endocannabinoid metabolism, our results do suggest enhanced lipid oxidation in ADC patients compared to those with SCC.

Because oxidative stress is closely related to inflammation, we decided to investigate potential correlations between oxPC levels, CRP, and WBC, which are used as inflammation markers. Moreover, CRP and WBC have been used as markers of the risk of incident lung cancer (Wong et al., 2020), and as predictors of early mortality (Isaksson et al., 2022) or survival (Bacha et al., 2017). We observed a correlation between WBC counts and the plasma levels of two oxPCs, PC 16:0/18:2; OH and PC 16:0/20:3; OH. Elevated neutrophil counts primarily drive the association between WBCs and NSCLC; therefore, further research considering different subtypes of WBCs (e.g., neutrophils or lymphocytes), especially that the neutrophil-to-lymphocyte ratio has been used as a prognostic factor in NSCLC patients (Bacha et al., 2017; Yang et al., 2021).

Both COPD and NSCLC are associated with oxidative stress and inflammation. Our results demonstrate elevated oxidative stress in NSCLC patients compared to that in COPD patients. Interestingly, Berg et al. (2018) observed that markers reflecting inflammation, endothelial activation, and extracellular matrix remodeling were elevated in the serum of patients with COPD compared to levels in lung cancer patients. The observed elevation of oxPC in NSCLC compared to that in COPD patients (our data), together with elevated levels of oxidative stress markers in NSCLC tissues (Gęgotek et al., 2016) and enhanced inflammation mediators in COPD patients (Berg et al., 2018), suggest that the observed alterations in oxPC levels in NSCLC subjects were related to oxidative stress rather than inflammation.

Considering the variety of factors influencing the activity and properties of oxPCs, the very precise interpretation of our results obtained is challenging. Instead, these results demonstrate a clear need to explore the involvement of oxPCs in NSCLC. Furthermore, because oxPCs are potent stimulators of endothelial barrier disruption, therapies that attenuate their activities can result in novel therapeutic tools for anti-cancer treatment. However, elevated oxidative stress is associated with several tumor types, and increased levels of oxPCs are associated with prostate (Ingram et al., 2021), pancreatic (Stevens et al., 2012) and neuroendocrine cancers (López-López et al., 2020). Therefore, oxPCs themselves cannot be used as potential biomarkers of NSCLC due to their lack of specificity. However, Philippova et al. (2019) pointed out that simultaneous determination of plasma levels of oxPCs and estimation of the detoxifying capacity of plasma to neutralize these lipids can significantly improve the diagnostic importance of oxPCs as a disease biomarker. In the context of our results, oxPCs might be rather considered as potential markers to distinguish between different NSCLC subtypes instead, however this requires more investigation, including the quantification of these lipids in plasma. Although, the implementation of oxPCs into the clinical diagnosis process requires their quantification in plasma samples, this will be very difficult due to the limited availability of standards of these substances, let alone labeled ones.

## 5 Conclusion

Our results revealed that the oxPC profiles of patients with NSCLC were different between plasma and tumor tissues. LCh-oxPCs were the predominant form in plasma (62% of all detected oxPCs), and SCh-oxPCs were the main fraction in tissue (94% and 70% of all detected oxPCs in the total tumor and dimensional tumor tissue analyses, respectively). OxPC levels were significantly elevated in the plasma of patients with NSCLC compared to those in COPD controls. They were also elevated in the tumor tissue of patients with NSCLC compared to levels in the non-cancerous adjacent lung tissue, with the lowest level of oxPCs observed in the peripheral part of the tumor. Moreover, a higher level of oxPCs was observed in ADC patients than in SCC patients compared to that in COPD controls. A higher level of oxPCs was also observed in the tumor tissue of both ADC and SCC patients than in the surrounding non-cancerous lung tissue, but this difference was more prominent in ADC patients. Circulating oxPC levels correlated with WBC count but not with CRP level, whereas tissue oxPC levels did not correlate with tumor size (MTV) or activity (SUV).

Finally, the elevation of oxPC levels in NSCLC relative to that in COPD patients, together with higher levels of oxidative stress markers in NSCLC (Gęgotek et al., 2016) and enhanced inflammation mediators (Berg et al., 2018) in COPD patients, suggest that the observed alterations in oxPC levels in NSCLC are more closely related to oxidative stress than inflammation.

The obtained results confirm the involvement of oxPCs in NSCLC. Given that oxPCs are a common hallmark of oxidative stress accompanying multiple cancer types and the high toxicity of SCh-oxPCs, their accumulation in NSCLC patients highlights their potential as therapeutic targets rather than biomarkers.

## Data Availability

The raw data supporting the conclusion of this article will be made available by the authors, without undue reservation.
