# Supplementary material for: Exploration of oxidized phosphocholine profile in non-small-cell lung cancer
Source: Front Mol Biosci. 2024 Jan 15;10:1279645. doi: 10.3389/fmolb.2023.1279645 (PMC10824250; doi:10.3389/fmolb.2023.1279645)
Supplement: Supplementary file 1 [file Table1.pdf]

Table 1S: The list of oxPCs used for the targeted data processing.

oxPC	Formula	Ion	Intensity* [counts]	Annotation of tissue oxPCs		Annotation of plasma oxPCs
				Mass error [ppm]	RT error [min]	MS/MS fragments
PC(16:0/4:0-CHO)	$C_{44}H_{81}NO_8P$	$[M+HCOO]^-$				
PC(16:0/4:0-CHO)	$C_{44}H_{81}NO_8P$	$[M+HCOO]^-$	587	-1.03	-0.09	
PC(16:0/5:0-CHO)	$C_{29}H_{56}NO_9P$	$[M+HCOO]^-$	1,446	-1.52	-0.04	
PC(16:0/6:0-CHO)	$C_{30}H_{58}NO_9P$	$[M+HCOO]^-$				
PC(16:0/6:0-CHO)	$C_{30}H_{58}NO_9P$	$[M+HCOO]^-$				
PC(16:0/7:0-CHO)	$C_{31}H_{60}NO_9P$	$[M+HCOO]^-$		-1.13	-0.04	
PC(16:0/7:0-CHO)	$C_{31}H_{60}NO_9P$	$[M+HCOO]^-$				
PC(16:0/7:0-CHO)	$C_{31}H_{60}NO_9P$	$[M+HCOO]^-$				
PC(16:0/7:0-CHO)	$C_{31}H_{60}NO_9P$	$[M+HCOO]^-$				
PC(16:0/9:0-CHO)	$C_{33}H_{67}NO_9P$	$[M+HCOO]^-$				694.4301; 648.4246; 634.4089; 171.1027
PC(16:0/9:0-CHO)	$C_{33}H_{67}NO_9P$	$[M+HCOO]^-$				
PC(16:0/10:0-CHO)	$C_{34}H_{66}NO_9P$	$[M+HCOO]^-$				708.4457; 662.4402; 648.4246; 185.1183
PC(16:0/11:0-CHO)	$C_{35}H_{68}NO_9P$	$[M+HCOO]^-$				722.4614; 676.4559; 662.4402; 199.1340
PC(16:0/11:0-CHO)	$C_{35}H_{68}NO_9P$	$[M+HCOO]^-$				
PC(16:0/8:1-CHO-OH)	$C_{32}H_{60}NO_{10}P$	$[M+HCOO]^-$				
PC(16:0/8:1-CHO-O)	$C_{32}H_{58}NO_{10}P$	$[M+HCOO]^-$				
PC(16:0/4:0-COOH)	$C_{28}H_{54}NO_9P$	$[M-H]^-$				
PC(16:0/5:0-COOH)	$C_{28}H_{54}NO_{10}P$	$[M-H]^-$	512	-0.14	-0.02	
PC(16:0/6:0-COOH)	$C_{30}H_{58}NO_{10}P$	$[M-H]^-$				
PC(16:0/7:0-COOH)	$C_{31}H_{60}NO_{10}P$	$[M-H]^-$	9,537	-0.16		634.3726; 575.2991; 171.0663; 139.0401; 95.0502
PC(16:0/7:0-COOH)	$C_{31}H_{60}NO_{10}P$	$[M-H]^-$	328	7.22	-0,05	
PC(16:0/7:0-COOH)	$C_{31}H_{60}NO_{10}P$	$[M-H]^-$				
PC(16:0/7:0-COOH)	$C_{31}H_{60}NO_{10}P$	$[M-H]^-$	1,180	-6.27	-0,08	
PC(16:0/9:0-COOH)	$C_{33}H_{64}NO_{10}P$	$[M-H]^-$				664.4195; 632.3933; 605.3460; 255.2331; 171.1027
PC(16:0/9:0-COOH)	$C_{33}H_{64}NO_{10}P$	$[M-H]^-$				

PC(16:0/9:0-COOH)	$C_{33}H_{64}NO_{10}P$	$[M-H]^-$				
PC(16:0/9:0-COOH)	$C_{33}H_{64}NO_{10}P$	$[M-H]^-$				
PC(16:0/9:0-COOH)	$C_{33}H_{64}NO_{10}P$	$[M-H]^-$				
PC(16:0/9:0-COOH)	$C_{33}H_{64}NO_{10}P$	$[M-H]^-$				
PC(16:0/9:0-COOH)	$C_{33}H_{64}NO_{10}P$	$[M-H]^-$				
PC(16:0/8:1-COOH-OH)	$C_{32}H_{60}NO_{11}P$	$[M-H]^-$				
PC(16:0/8:1-COOH-O)	$C_{32}H_{58}NO_{11}P$	$[M-H]^-$				
PC(16:0/20:4-OH)	$C_{44}H_{81}NO_9P$	$[M+HCOO]^-$		-3.76	-0,10	
PC(16:0/20:4-OH)	$C_{44}H_{81}NO_9P$	$[M+HCOO]^-$	135,971	-3.26		843.5631; 825.5526; 319.22783; 301.2173; 275.23803; 255.2331
PC(18:0/20:4-OH)	$C_{46}H_{85}NO_9P$	$[M+HCOO]^-$	133,802	-0.60		871.5944; 853.5839; 319.22783; 301.2173; 275.23803; 283.2642
PC(16:0/22:6-OH)	$C_{46}H_{81}NO_9P$	$[M+HCOO]^-$				867.5631; 849.5526; 343.2278; 325.2173; 299.238; 255.2331
PC(16:0/20:3-OH)	$C_{44}H_{83}NO_9P$	$[M+HCOO]^-$	87,985	-2.25		845.5787; 827.5682; 321.2435; 303.2330; 255.2331
PC(16:0/18:2-OH)	$C_{42}H_{81}NO_9P$	$[M+HCOO]^-$	104,087<br>4,227	-4.00<br>0.26<br>4.78	-0.10<br>-0.03	847.5946; 829.5839; 295.2278; 277.2173; 255.2331
PC(18:0/18:2-OH)	$C_{44}H_{85}NO_9P$	$[M+HCOO]^-$	224,894	2.62		819.5631; 801.5526; 295.2278; 277.2173; 283.2642
PC(16:0/4:0-(OH)2)	$C_{28}H_{56}NO_{10}P$	$[M+HCOO]^-$				
PC(16:0/5:0-(OH)2)	$C_{29}H_{58}NO_{10}P$	$[M+HCOO]^-$				
PC(16:0/20:4-OOH)	$C_{44}H_{81}NO_{10}P$	$[M+HCOO]^-$				
PC(18:0/20:4-OOH)	$C_{46}H_{85}NO_{10}P$	$[M+HCOO]^-$	97,103	-0.36		859.5580; 841.5475; 825.5531; 326.2234; 308.2234; 292.2185; 282.2336; 255.2331
PC(18:0/20:4-OOH)	$C_{46}H_{85}NO_{10}P$	$[M+HCOO]^-$				
PC(18:0/20:4-OOH)	$C_{46}H_{85}NO_{10}P$	$[M+HCOO]^-$				

The oxPCs with listed MS/MS fragments were annotated in plasma samples, and their annotation must be confirmed with structural information. * averaged intensity

Table 2S: Differences in oxPC profiles of NSCLC patients and COPD controls in plasma samples (Panel A) and non-cancerous lung tissue and tumor tissue from NSCLC patients (Panel B) for all, female and male patients.

A) Relative plasma oxPC abundance between NSCLC patients and COPD controls				
**oxPC**	**raw *p*-value**	**all**	**female**	**male**
			[%]	
PC 16:0/20:3;OH	2.46E-14	NSCLC	NSCLC	NSCLC
PC 16:0/18:2;OH	1.95E-13	179	125	197
PC 16:0/20:4;OH	2.36E-11	88	56	102
PC 18:0/20:4;OH	3.23E-08	54	36	66
PC 18:0/20:4;OOH	1.75E-02	14	1	24
PC 18:0/18:2;OH	2.44E-02	17	8	29
B) Relative oxPC levels between healthy lung tissue and tumor tissue from NSCLC patients				
**oxPC**	**raw *p*-value**	**change**	**female**	**male**
			[%]	
PC 16:0/7:0;COOH iso4	6.61E-05	87	55	99
PC 16:0/7:0;COOH iso2	8.66E-05	-10	-14	-9
PC 16:0/18:2;OH iso2	1.59E-03	76	50	82
PC 16:0/4:0;CHO iso2	5.62E-03	65	43	70
PC 16:0/5:0;CHO	7.79E-03	73	67	74
PC 16:0/5:0;COOH	2.05E-02	92	77	96

Raw p-value computed with the Mann-Whitney unpaired test for plasma comparisons and paired test for tissue comparisons. Corrected p-value was calculated employing Benjamini-Hochberg FDR correction.

The positive value of the change indicates that the average amount of oxPC is higher in plasma of cancer patients than in controls, and higher in tumor tissue than in healthy lung tissue, while a negative value indicates that the average amount of oxPC is lower in plasma of cancer patients than in controls, and lower in tumor tissue than in healthy lung tissue.
